# The Plasma Neurofilament Light Chain, Brain-Derived Neurotrophic Factor, and Risk of Depression in Chronic Hemodialysis Patients

**DOI:** 10.3390/biomedicines12010103

**Published:** 2024-01-04

**Authors:** Martyna Stanisławska, Maja Roman, Michał Nowicki

**Affiliations:** Department of Nephrology, Hypertension and Kidney Transplantation, Central University Hospital, Medical University of Lodz, Pomorska 251, 92-213 Lodz, Poland; martynastanislawska97@gmail.com (M.S.); maja.roman@stud.umed.lodz.pl (M.R.)

**Keywords:** chronic hemodialysis patients, depression, neurofilament light chain, brain-derived neurotrophic factor, hemodialysis

## Abstract

Introduction: Depression is highly prevalent among hemodialysis patients. Understanding the relationship between the plasma neurofilament light chain (NfL) and brain-derived neurotrophic factor (BDNF) may help us to better understand the mechanisms of depression. This study determined their impact, alongside that of other factors, on the risk of depression in hemodialysis patients. Methods: The study enrolled 82 patients undergoing chronic hemodialysis. Serum NfL, BDNF, uric acid, urea, potassium, calcium, phosphorus, intact parathyroid hormone, and C-reactive protein (CRP) levels were measured. The patients completed the Beck Depression Inventory (BDI). Blood pressure values, body mass before and after hemodialysis, and weekly duration of hemodialysis in hours were assessed. For 19-month survival analysis, the patients were stratified according to baseline BDI scores. Results: Based on the BDI score, 18.3% of the patients had an increased risk of depression. Lower scores were associated with significantly longer duration of hemodialysis treatment (37.5 (25–57) 24 (14–37) months, *p* = 0.01). Within the 19-month survival analysis, 31.7% of patients died. The patients with BDI scores above the median had significantly lower survival than those below the median (log-rank test *p* = 0.02). No significant differences in serum BDNF levels (192.7 [125.2–278.2]; 207.7 [142.8–265.8] pg/mL, *p* = 0.40), or NfL concentrations (1431.5 [1182.6–1625.7]; 1494.6 [1335.7–1667] kDa, *p* = 0.52) were found between patients with lower and higher risk of depression. Patients with BDI scores above the median had significantly higher levels of CRP (9.6 [4.4–14]) than those with scores below the median (3.6 [2.2–7.5], *p* = 0.01). A significant positive correlation was found between the BDI score and serum CRP level (r = 0.38, *p* = 0.01). A significant negative correlation was observed between the BDI score and URR% value (r = −0.36, *p* = 0.02). Conclusions: Patients with lower BDI scores had a longer dialysis duration, indicating a potential negative association between depression risk and length of dialysis treatment. Neither serum NfL nor BDNF levels can serve as markers of depression risk in the dialysis population.

## 1. Introduction

Depression is the most common mental disorder in patients with end-stage kidney disease (ESKD); according to various estimates, it affects from a quarter to a half of chronic dialysis patients [1,2]. The discrepancy in these epidemiological data may be due to the similarity between depressive and uremic syndromes, which may occur in parallel in most patients with ESKD. Several studies have linked depression to mortality in ESKD, making early diagnosis and treatment of this disease necessary [1].

Despite a plethora of research, the specific pathomechanism of depression has not been fully elucidated in patients with ESKD. It is believed that hemodialysis patients often experience a broad range of health problems, such as fatigue, chronic pain, and limitations in daily activities, all of which can contribute to a depressed mood [1]. Furthermore, dialysis-related stressors, such as frequent hospital visits, harsh lifestyle constraints, and treatment complexity, add to the stress burden and induce depressive symptoms [3]. Social isolation and lack of support are additional factors that can affect the development of depression in hemodialysis patients. Moreover, financial difficulties associated with the challenges of finding employment due to a severe health condition can cause significant stress and contribute to mental health problems [4]. Other risk factors for depression in hemodialysis patients include sleep disturbances, medication side effects, body image issues, and limited physical functioning [5].

In relation to the numerous consequences of depression, such as poor cooperation with medical personnel, reduced motivation for treatment, non-compliance with dietary guidelines, and even an increased risk of suicide, extensive research has been conducted to search for new depression biomarkers in this population. Plasma brain-derived neurotrophic factor (BDNF) and neurofilament light chain (NfL) concentration may be potential biomarker candidates because their increased levels have been detected in non-dialysis patients with a history of attempted suicide [6,7]. The neurobiological mechanisms underlying depressive and anxiety disorders have been shown to involve an altered BDNF signaling pathway in subjects with depression. In addition, patients with depression showed markedly lower plasma BDNF levels than did healthy subjects. Many studies have suggested that BDNF is critically reduced in mood disorders and plays an important role in the response to antidepressant therapy. BDNF is found in nearly all brain regions, neurons, glia, and vascular compartments, and is involved in many central activities, including development, neurogenesis, glycogenesis, synaptogenesis, neuroprotection, memory and cognition. Recent studies suggest that BDNF plays a crucial role in responding to oxidative stress, which is significant in the context of inflammatory conditions observed in hemodialysis patients [8,9]. The selection of BDNF as a marker in the study allows for investigating the relationship between BDNF levels and neurobiological, metabolic, and inflammatory factors specific to patients undergoing hemodialysis [10]. C-reactive protein, as an indicator of systemic inflammatory response, allows for the identification of potential connections between inflammation and depression in hemodialysis patients. The integrated approach, assessing both BDNF and CRP, aims to offer a comprehensive view of the intricate interaction among oxidative stress, inflammatory conditions, and depression in these patients.

NfL, on the other hand, is a neuronal cytoplasmic protein highly expressed in large-caliber myelinated axons. Its levels increase in the cerebrospinal fluid and blood in proportion to the degree of axonal damage in a variety of neurological disorders, including inflammatory, neurodegenerative, traumatic and cerebrovascular diseases. Increased levels of NfL may play a protective role in diseases with neuronal damage, as it is likely involved in their regeneration. Blood NfL has been shown to be a biomarker of axonal damage in a variety of neurological disorders, including major depressive disorder. In the context of depression in patients undergoing hemodialysis, the choice of NfL as a marker gains additional significance. Its role in indicating axonal damage aligns with the potential neurological complications observed in this patient group [11]. Thus, although both BDNF and NfL can serve as potential markers of depression, the concept remains debatable owing to diverging research results.

The aim of our study was to assess whether serum levels of NfL and BDNF are associated with the risk of depression in ESKD patients. In this study, we considered the significant impact of various other factors, such as uric acid, potassium, calcium, phosphorus, and PTH, on neurological and psychological functions. Incorporating the potential influence of these factors is essential, because uric acid may affect mental well-being through neuroinflammatory processes [12], and the potassium level, along with the balance between calcium and phosphorus, may influence mood and the functioning of the nervous system [13,14]. This comprehensive approach allows for a more nuanced exploration of the connections between different components and depressive symptoms in patients undergoing hemodialysis.

## 2. Material and Methods

The cross-sectional part of the study was conducted from May 2021 to November 2022, and patients were followed up for survival analysis for up to 19 months. Some 82 adult chronic hemodialysis patients with at least a 4-week history of chronic hemodialysis treatment were included. Enrolled patients were over 18 years of age and were treated with conventional hemodialysis with a high-flux dialyzer three times per week for at least 12 h weekly. Written informed consent was obtained from all participants, and the study was conducted in accordance with the Declaration of Helsinki. The study protocol was approved by the local ethics committee.

The exclusion criteria were a previous diagnosis and a history or ongoing treatment of depression, any other mental or motor disorder that would preclude any study procedure, acute inflammation (serum C-reactive protein > 15 mg/L), or any clinical signs of infection.

The baseline clinical and laboratory characteristics of the study population are shown in Table 1.

Blood samples were obtained immediately before the commencement of the mid-week (i.e., scheduled for either Wednesday or Thursday) hemodialysis session from all participants to determine blood hemoglobin and plasma concentrations of neurofilament light polypeptide (NfL), brain-derived neurotrophic factor (BDNF), uric acid, potassium, calcium, phosphorus, intact parathyroid hormone, and C-reactive protein (CRP). Serum urea levels were measured before and after dialysis to calculate the urea reduction ratio as a measure of dialysis adequacy. The urea reduction ratio was calculated using the formula = (C0 − Ct)/C0, utilizing known concentrations of urea before (C0) and after the HD procedure (Ct) [15].

The plasma BDNF concentration was measured using a commercially available ELISA kit BDNF manufactured by RayBiotech, Inc. (Norcross, GA, USA). The substrate solutions were prepared according to the manufacturer’s instructions.

The concentration of NfL was evaluated using a neurofilament light polypeptide (NEFL) ELISA Kit (Fisher Scientific, Hampton, NH, USA), with a sensitivity of 6.2 pg/mL.

Blood was drawn into two EDTA tubes. The total volume of blood obtained was 10 mL. EDTA tubes were immediately centrifuged at 2500 rpm and 4 °C, and the plasma was frozen at −80 °C. Laboratory assessments were performed in batches at local research lab. All other parameters were measured in a local laboratory using routine automated laboratory methods.

Each patient completed the Beck Depression Inventory (BDI) at the beginning of the hemodialysis session [16]. Depending on their health condition, most patients completed the questionnaire independently, while 15% of patients required minimal assistance in reading a choice of questions and answers. The Beck Depression Inventory (BDI) score is commonly used as a screening tool for depression. Its significance lies in its ability to precisely measure various aspects of depression, which is crucial for hemodialysis patients, where depressive symptoms can have diverse manifestations. The reliability of BDI is based on its extensive use and standardization in depression research over the years. It has confirmed psychometric validations, indicating its effectiveness and clarity in measuring depression. The BDI cut-off point for the diagnosis of depression is a score of 14 points or more [16].

In addition, systolic and diastolic blood pressures before and after the same hemodialysis, weekly duration of hemodialysis in hours, and body mass before and after hemodialysis were recorded. Blood pressure in the right arm was measured three times using a DINAMAP 1846 SX automatic oscillometric device (Critikon, Inc., Tampa, FL, USA). Blood pressure was calculated for further analysis as the mean of three consecutive readings.

An additional survival analysis was performed to assess the relationship between the BDI scores and survival. The Cox proportional hazards model was used for the survival analysis. Survival analysis results are presented using a survival curve.

Statistical analysis was performed using Statistica ver. 13.1 PL software (StatSoft Inc., Tulsa, OK, USA). Graphs were plotted using MS Excel, The version is 2311,(Microsoft Corp., Redmond, WA, USA) and Statistica software. Results are presented as mean ± standard deviation (SD) or median and 25–75% interquartile range (IQR), depending on the normality of the distribution of each variable assessed with the Shapiro–Wilk test. A *t*-test was used to compare the means between two independent groups of normally distributed variables. ANOVA and post hoc tests were used to compare to three groups divided into tertiles. The chi-squared test was used to compare categorical data. Linear correlations between pairs of variables were assessed using Pearson’s or Spearman’s method, depending on the distribution of each variable.

## 3. Results

Nearly one fifth of the dialysis patients (18.3%) had a BDI score of 14 points, indicating an increased risk of depression. For further analysis, the study population was divided into two subgroups based on the median BDI score (seven points).

Table 2 presents the main results of the study. Patients who scored lower on the BDI were dialyzed for a significantly longer time than those who scored higher (37.5 (25–57) vs. 24 (14–37) months, respectively, *p* = 0.01).

Additionally, in the patients who scored above the median, significantly higher values of C-reactive protein were seen compared to the group of patients with a BDI score below the median (9.6 (4.4–14) and 3.6 (2.2–7.5) points, respectively; *p* = 0.01).

**Figure 1:**
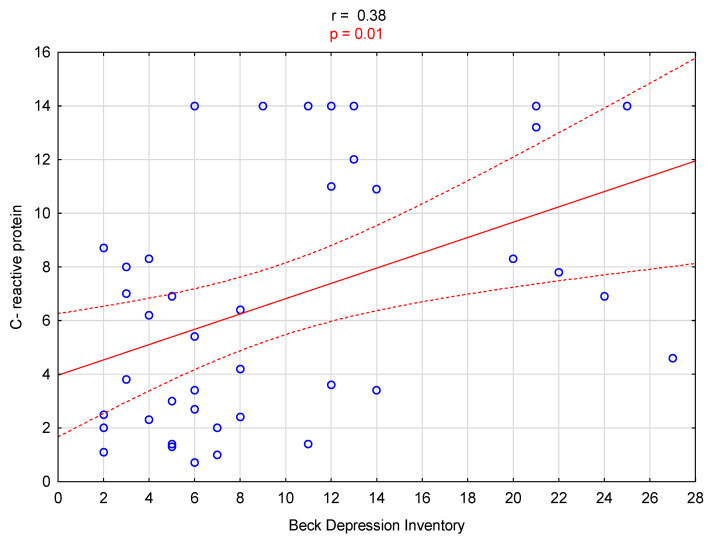
Graph illustrating the correlation between C-reactive protein levels and Beck Depression Inventory scores.

**Figure 2:**
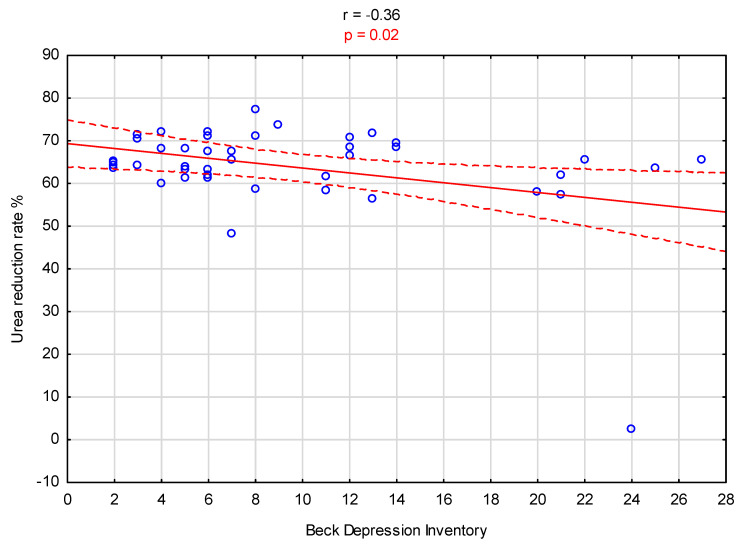
Graph illustrating the correlation between urea reduction rate % levels and Beck Depression Inventory scores.

A significant positive correlation was observed between the BDI score and the serum CRP level (r = 0.38, *p* = 0.01; Figure 1). Additionally, there was a significant negative correlation between the BDI score and the urea reduction ratio (URR%) value (r = −0.36, *p* = 0.02; Figure 2).

No significant correlations were found between the BDI score and duration of dialysis therapy (r = −0.27, *p* = 0.09) and post-dialysis urea concentration (r = 0.27, *p* = 0.09). No significant correlation was observed between the BDI score and interdialytic weight gain (r = 0.28, *p* = 0.07), as well as between the time dedicated to dialysis sessions per week (r = 0.26, *p* = 0.10).

The linear regression model included the dependent variable, BDI score, and independent variables such as blood hemoglobin, plasma concentrations of neurofilament light polypeptide and brain-derived neurotrophic factor, uric acid, potassium, calcium, phosphorus, intact parathyroid hormone, CRP, serum urea level, and urea reduction ratio. A statistically significant relationship was found between the BDI score and serum CRP level. Regression analysis revealed that the results accounted for 17% of the variance in the dependent variable (β = 0.417; *p* = 0.01; R^2^ = 0.17).

**Figure 3:**
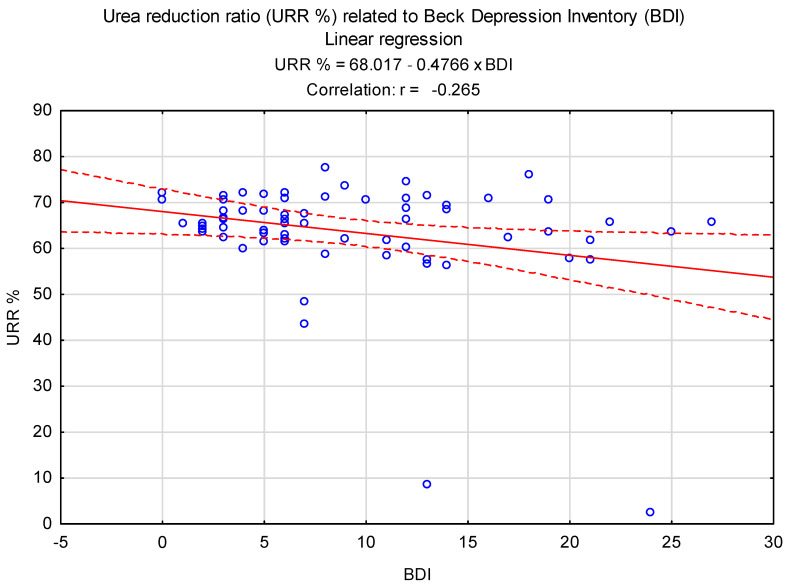
Linear regression plot of URR% relative to BDI score values.

**Figure 4:**
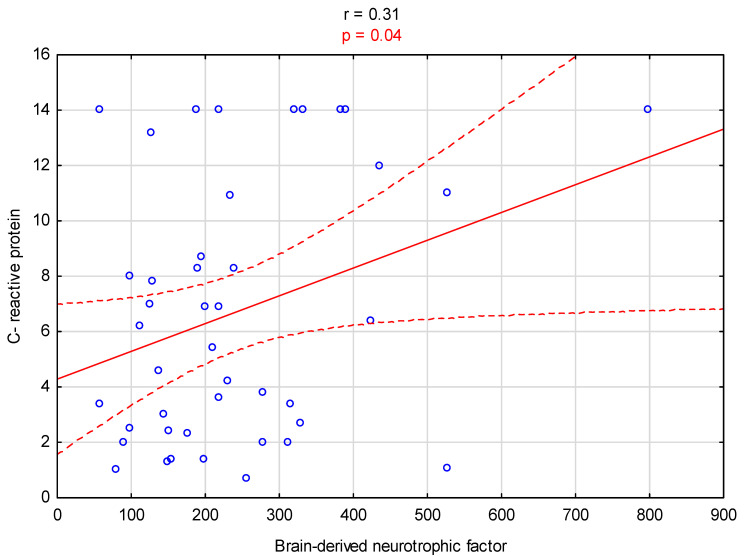
Graph illustrating the correlation between C-reactive protein and brain-derived neurotrophic factor levels.

Additionally, a significant negative relationship was observed between BDI and URR% levels (β = −0.265, *p* = 0.03; R^2^ = 0.07, Figure 3). A significant positive correlation was observed between CRP levels and the BDNF concentration in the serum of patients (r = 0.31, *p* = 0.04, Figure 4).

In addition, no significant correlation was observed between the plasma NfL level and patient age (r = 0.26, *p* = 0.10).

**Figure 5:**
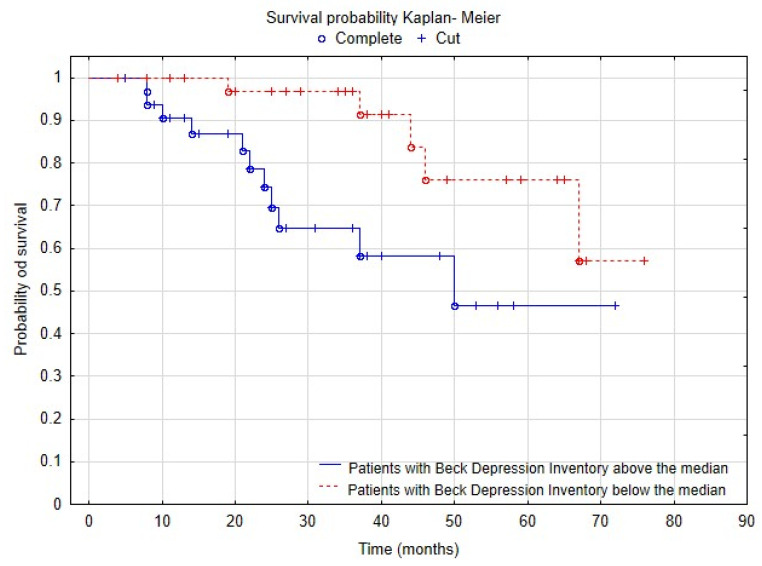
A Kaplan–Meier survival analysis plot.

A survival analysis was conducted, with a total of 82 observations. During the 19-month-long observation, 26 patients died, accounting for 31.7% of the initial study population. The analysis was also performed with the sample divided into two groups based on BDI scores. The first group consisted of patients with scores below the median, while the second group consisted of patients with scores above the median. The total number of valid observations was 72, including 18 patients who died (having had complete observations). There were 57 patients in the group below the median, and 13 of them died, representing 22.8% of the group. In the group above the median, there were 15 patients, and 5 of them died, representing 33.3% of that group. The probability of survival among patients with higher BDI scores was significantly lower than that among patients with scores below the median (log-rank test; *p* = 0.02). The results are shown in Figure 5.

## 4. Discussion

In contrast with our initial hypothesis, the study did not show any significant relationship between plasma levels of NfL and BDNF and the presence of patient-reported depressive symptoms. Our study, however, confirmed a high risk of depression among hemodialysis patients, and found that the patients with lower depression risk have better survival.

The association of depressive symptoms with BDNF levels has been investigated in several studies [17,18,19], and our results contrast with some of them. Eraldemir et al. [17] found a significant negative correlation between the BDI score and serum BDNF levels in hemodialysis patients. The reasons for these different results may include differences in the modality of the dialysis therapy. In our study, only individuals undergoing hemodialysis were included, whereas in the cited study, both patients undergoing peritoneal dialysis and hemodialysis were investigated. However, our results corroborate the findings of Lee et al. [19], who were unable to find any significant correlation between BDNF levels and BDI scores in patients undergoing hemodialysis. Cohort studies on the long-term impact of hemodialysis on BDNF levels are limited, but some analyses suggest potential changes in these levels as hemodialysis therapy progresses [20]. It is worth considering that the dynamics of these changes may be subtle, and require a longer observation period.

Since neurofilament light chain (NfL) is a neuronal cytoskeleton protein, its blood serum levels can serve as a surrogate marker of neuronal damage. In recent years, several studies have investigated NfL levels in patients undergoing hemodialysis. In a study by Chen et al. (2022) [21], the serum NfL level was on average 3.7 pg/mL higher in hemodialysis patients than in healthy subjects. There have also been studies confirming a higher serum level of NfL in patients with major depression than in the control group [22]. These results suggest that neuronal damage may be associated with depression. However, no study has confirmed this finding in relation to the risk of depression.

In our study, we did not find any significant correlation between the level of NfL and BDI score in hemodialysis patients. To the best of our knowledge, no previous study has evaluated serum NfL levels in hemodialysis patients in relation to depression.

The lack of significant differences in NfL and BDNF levels in our study may be attributed to several factors. Firstly, the heterogeneity in clinical characteristics among hemodialysis patients, such as their stage of kidney disease, comorbidities, and treatment history, could contribute to diverse outcomes. Secondly, individual genetic differences in the metabolism of neurotropins and NfL protein may influence the levels of these substances, without necessarily correlating with the presence of depression.

Our study revealed that serum C-reactive protein (CRP) levels were positively associated with higher BDI scores in hemodialysis patients. It needs to be underlined that the patients with CRP values > 15 mg/L or any symptoms indicating infection were excluded from our study. This finding may suggest that depression could be a comorbidity factor in patients undergoing hemodialysis, and is closely related to the state of so called “micro-inflammation” [23].

High CRP levels in hemodialysis patients with depression may be due to various reasons. Several potential factors may contribute to the elevated serum CRP levels in most of this population. First, chronic stress, often experienced by individuals with depression, can activate the immune system and trigger inflammation, resulting in elevated CRP levels. In addition, sleep disorders such as insomnia or poor sleep quality, which are common in patients with depression, can also affect CRP levels. Unhealthy eating habits, which are common in people with depression, can also contribute to elevated CRP levels. In addition, low levels of physical activity, often associated with depression and hemodialysis, can contribute to inflammation and elevated CRP levels.

In the case of hemodialysis patients, who are already predisposed to inflammation, the elevation in C-reactive protein (CRP) levels may exhibit heightened discernibility by healthcare professionals responsible for monitoring patients. Hemodialyzed patients frequently present with elevated baseline levels of inflammatory markers due to their underlying renal disease and the dialysis procedure itself, thereby leading to an augmented CRP response. Many studies have shown a relationship between inflammation (measured by CRP level) and depression in hemodialysis patients (Dogan et al. (2005) [24]). This could have important clinical implications, allowing doctors to gain a better understanding and monitor potential effects of inflammation in hemodialysis patients, particularly regarding depression. Elevated CRP levels may serve as an indicator for medical professionals, signaling a potential risk of depression in these patients.

Additionally, our study revealed a statistically significant negative correlation between urea reduction ratio (URR%) and Beck Depression Inventory (BDI) scores. Depression is associated with neuroendocrine dysfunction and increased sympathetic activity, which can result in vasoconstriction and reduced blood flow to dialysis access sites. Insufficient blood flow during dialysis can hinder the removal of urea and other metabolic waste products, negatively affecting the URR% [25].

Other studies have also confirmed a negative correlation between URR% and depression in hemodialysis patients [26]. A decrease in URR% in hemodialysis patients with depression is important, as it means that fewer toxins are removed from the body, increasing the risk of complications such as poisoning. Furthermore, a decrease in URR% may lead to a worse quality of life and an increased risk of hospitalization and death. Therefore, we may postulate that treating physicians should closely monitor dialysis patients with depression for a decrease in URR%, and modify renal replacement therapy if possible.

In our study, we also assessed the relationship between PTH levels and depression, since studies postulate that PTH serum levels are higher in hemodialysis patients with depression compared to those without depression [27]. However, our study did not show any significant correlation between PTH concentration and BDI score (r = −0.09, *p* = 0.56). Similar results were obtained by Bassola et al. (2010) [28], who also confirmed a lack of association between parathyroid hormone level and depression in hemodialysis patients.

In our study, after dividing the group by the median BDI score (7 points), we observed a significant difference in the hemodialysis vintage. Patients with higher BDI scores were dialyzed for an average of 14 months less than those with lower BDI scores. Interestingly, this relationship was not observed in other studies conducted so far in hemodialysis patients. In a study by Wu et al. (2022) [29], even an inverse relationship was observed, i.e., as the duration of dialysis increased, the level of depression also increased. Another study also found no association between these parameters (Hsu 2009) [30].

Our results allow us to speculate that patients who are dialyzed for a shorter period may be less willing to undergo drastic changes in their lifestyle, which may affect their mental well-being. In contrast, patients who are dialyzed for a longer period may be more likely to reconcile with their disease. Therefore, it becomes important to inform medical staff about the significance of providing psychological assistance to patients right from the beginning of therapy. Such care includes both psychological support and education on coping with the emotional challenges associated with hemodialysis. Offering early psychological support can significantly enhance quality of life for patients during hemodialysis.

Although the follow-up period in our study was relatively short we conducted an additional survival analysis, which showed that patients who had higher levels of depressive symptoms had a significantly higher risk of dying earlier. Interestingly, survival studies in hemodialyzed patients that compared patients with and without depression showed diverse results. While some studies suggested that patients with depression showed much worse outcomes in terms of survival and cardiovascular risk because they were more prone to hospitalizations and death [31], other studies did not confirm such a relationship, and found no significant differences in survival between patients with and without depression [32,33]. Our study sheds new light on existing knowledge, suggesting that even a brief observation period can have practical implications for identifying the risk of premature death. In the context of comprehensive management of hemodialysis patients, our findings underscore the importance of focusing on effective depression treatment, which may impact life expectancy.

Our study had several limitations. First, our study group might not have been large enough to create sufficiently large subgroups after division into tertiles or quartiles for extended analysis. Second, hemodialysis patients are undergoing polypharmacotherapy, and some drugs may affect the laboratory parameters that we analyzed. The limitations of our study also arise from its cross-sectional nature, precluding the assessment of dynamic changes over time. It is important to note that while a cross-sectional study does not allow tracking changes over time, it can provide baseline data and preliminary insights, serving as a starting point for future, more comprehensive longitudinal investigations.

In conclusion, this study showed that the prevalence of self-assessed symptoms of depression in chronic hemodialysis patients with end-stage kidney disease is not related to the levels of plasma BDNF, NfL, and PTH. Therefore, these serum parameters may not serve as useful biomarkers of depression in the dialysis population. However, the study showed a significant negative correlation between URR% and BDI score, which may suggest that the efficiency of dialysis may affect the development of depression in these patients. In addition, our study showed significantly higher mortality in the group of patients with higher severity of depressive symptoms, suggesting that depression may be a risk factor for accelerated mortality in this population.

## Figures and Tables

**Table 1:** The clinical and biochemical characteristics of hemodialysis patients.

Variable	Mean ± SD or Median [Q1–Q3]
Women, *n* (%)	37 (43.9%)
Men, *n* (%)	47 (56.1%)
Age (years), Median [IQR, 25–75%]	68 [56–74]
Time on dialysis (months), Median [IQR, 25–75%]	26.5 [15–41]
Beck Depression Inventory, BDI score, Median [IQR, 25–75%]	7 [4–13]
Serum brain-derived neurotrophic factor, BDNF (pg/mL), Median [IQR, 25–75%]	211.7 [141.4–312]
Serum neurofilament light protein, NfL (kDa), Median [IQR, 25–75%]	1462.7 [1250–1672.5]
Body mass index (kg/m^2^), Mean ± SD	25 ± 4.8
Body mass before dialysis (kg), Mean ± SD	74.2 ± 15
Body mass gain between midweek dialysis sessions (kg), Mean ± SD	2.1 ± 1.1
Duration of dialysis per week (hours), Mean ± SD	10.2 ± 1.4
Blood hemoglobin (g/dL), Mean ± SD	10.5 ± 1.8
Serum urea before dialysis (mmol/L), Mean ± SD	23.4 ± 5.9
Serum urea after dialysis (mmol/L), Mean ± SD	8.4 ± 3.2
Urea reduction ratio in hemodialysis, URR%, Mean ± SD	63.5 ± 11.8
Serum phosphorus (mmol/L), Mean ± SD	1.8 ± 0.6
Serum calcium (mmol/L), Mean ± SD	2.5 ± 2.3
Serum parathyroid hormone (pg/mL), Mean ± SD	50.2 ± 50.6
Serum C-reactive protein (mg/L), Mean ± SD	8.4 ± 9.1

**Table 2:** Division of study population into two subgroups based on median BDI score.

Variable	Mean ± SD or Median [Q1–Q3] in Patients Who Score BDI Values below the Median	Mean ± SD or Median [Q1–Q3] in Patients Who Score BDI Values above the Median	*p*
Age (years)	68.5 [56–75]	66 [57–73]	0.91
Time on dialysis (months)	37.5 [25–57]	24 [14–37]	0.01
Serum brain-derived neurotrophic factor, BDNF (pg/mL)	192.7 [125.2–278.2]	207.7 [142.8–265.8]	0.40
Serum neurofilament light protein, NfL (kDa)	1431.5 [1182.6–1625.7]	1494.6 [1335.7–1667]	0.52
Body mass before dialysis (kg)	71.7 [61.4–78]	75 [62.7–79.9]	0.25
Body mass gain between midweek dialysis sessions (kg)	1.8 ± 0.9	2.2 ± 1.1	0.26
Duration of dialysis per week (hours)	10.2 ± 1.2	10.4 ± 1.2	0.56
Blood hemoglobin (g/dL)	10.8 [9.9–11.5]	10.8 [10.2–11.4]	0.63
Serum urea before dialysis (mmol/L)	22.3 [18.6–27.8]	23.2 [20–26.8]	0.84
Serum urea after dialysis (mmol/L)	7.7 [6–9.5]	8.1 [6.8–8.8]	0.41
Urea reduction ratio in hemodialysis, URR%	65.5 [63.1–70.4]	64.7 [58.4–70.7]	0.14
Serum phosphorus (mmol/L)	1.9 ± 0.6	1.9 ± 0.6	0.97
Serum calcium (mmol/L)	2.3 [2.1–2.4]	2.3 [2.1–2.3]	0.35
Serum parathyroid hormone (pg/mL)	36.4 [19.3–69]	42.4 [12.2–61.7]	0.52
Serum C-reactive protein (mg/L)	3.6 [2.2–7.5]	9.6 [4.4–14]	0.01

## Data Availability

Data is unavailable due to privacy or ethical restrictions.
